# Expression of the Cholecystokinin-B Receptor in Neoplastic Gastric Cells

**DOI:** 10.1007/s12672-017-0311-8

**Published:** 2017-10-04

**Authors:** Patricia Mjønes, Ivar S. Nordrum, Øystein Sørdal, Liv Sagatun, Reidar Fossmark, Arne Sandvik, Helge L. Waldum

**Affiliations:** 1 Department of Cancer Research and Molecular Medicine, Faculty of Medicine and Health Sciences, Norwegian University of Science and Technology, Trondheim, Norway; 2 Department of Pathology, St Olav’s Hospital—Trondheim University Hospital, Trondheim, Norway; 3 Department of Laboratory Medicine, Children’s and Woman’s Health, NTNU, Trondheim, Norway; 4 Department of Gastroenterology and Hepatology, St Olav’s Hospital—Trondheim University Hospital, Trondheim, Norway

**Keywords:** Gastrin receptor, Cholecystokinin-2 receptor, CCK2R, Cholecystokinin-B receptor, CCKBR, Neuroendocrine

## Abstract

Gastric cancer is an important disease due to its high mortality. Despite the decline in frequency, most cases are discovered late in its course, and most of the cancer patients die within a few years of diagnosis. In addition to *Helicobacter pylori* gastritis, gastrin is considered an important factor in the development of this disease, and thus, cholecystokinin-B receptor (CCKBR) becomes of interest. The aim of our study was to explore whether CCKBR is expressed in stomach cancers. Thirty-seven tumors from 19 men and 18 women diagnosed with either adenocarcinoma or neuroendocrine neoplasm (NENs) were included in this study. The tumors were classified into 29 adenocarcinomas and eight NENs. Immunohistochemistry with antibodies against chromogranin A (CgA), synaptophysin and CCKBR, and in situ hybridization with probes against CgA, CCKBR and histidine decarboxylase were used to further explore these tumors. Thirty-three (89%) of the tumors expressed CCKBR protein, whereas only 20 (54%) of all tumors expressed CCKBR mRNA. Of the 20 tumors expressing CCKBR mRNA, eight were NENs and 12 were adenocarcinoma. The highest amount of CCKBR was expressed in NEN. Interestingly, a high degree of co-expression of CCKBR and CgA was observed when the two markers were examined together with in situ hybridization. In conclusion, we found that all eight NENs expressed CCKBR and neuroendocrine markers in a majority of tumor cells. The same markers were also expressed in a proportion of adenocarcinomas supporting the view that gastrin is important in the development of gastric cancer.

## Introduction

Gastric cancer is one of the leading causes of cancer deaths worldwide and is ranked as the fifth most common malignancy [1]. Despite the decline in prevalence over the last decades, most cases are discovered at an advanced stage, and subsequently most of the cancer patients die within a few years of diagnosis. There are many ways of classifying gastric cancer, but Lauréns classification into diffuse, intestinal and indeterminate/mixed types is commonly applied. The various subtypes do not appear to transform into other, suggesting important biological discrepancies between the two [2, 3]. In addition, the intestinal type continues to decline in incidence, whereas the incidence of the diffuse type remains constant across the various populations, supporting this view [3].

A certain number of gastric carcinomas express neuroendocrine (NE) markers [4, 5]. A few studies even suggest that gastric carcinomas of the diffuse type originate from enterochromaffin-like (ECL) cells by dedifferentiation [6, 7]. Co-occurrence of neuroendocrine tumors (NETs) and gastric carcinomas in patients with chronic atrophic gastritis (CAG) is also an indication of a close relationship between the two entities [8]. A number of gastric carcinomas of the intestinal type also express NE markers, but not as commonly as with the diffuse type. In some patients with gastric carcinoma, there are elevated gastrin levels [9, 10], incriminating gastrin as a driving force in the development of these cancers.

There are three types of neuroendocrine tumors (NETs) in the stomach. Type 1 is the most common accounting for approximately 80% of cases and is associated with CAG [11, 12]. ECL cells of the gastric mucosa are known to express cholecystokinin-B receptors (CCKBR), also known as gastrin receptor or cholecystokinin-2 receptors (CCK2R) [13]. Originating from ECL cells, NET type 1 is thought to express CCKBR. Studies done on patients with CAG and NETs treated with the CCKBR antagonist netazepide, have shown promising results in terms of decrease in tumor size and in some patients complete remission [14, 15]. The effect of gastrin is mediated by the binding of gastrin to its receptor CCKBR. CCKBR is a seven-transmembrane G protein-coupled receptor expressed in the proximal two thirds of the stomach [16]. The presence of CCKBR on the ECL cell and its role in acid secretion is undisputed [17], but there is controversy with regard to the expression of this receptor on the parietal and other cells.

Even though the incidence of stomach cancer is decreasing, it is still an important disease due to its high mortality. The reduced occurrence of stomach cancer has mainly affected adenocarcinoma of the intestinal type, the incidence of the diffuse type remaining largely unchanged [18, 19]. The association between *Helicobacter pylori* infection and gastric adenocarcinoma is well established [20], and thus, the reduction in adenocarcinoma of the intestinal type is partly explained by eradication of the bacterium as well as reduced incidence of *Helicobacter pylori* infection. Hypoacidity and hypergastrinemia are also factors associated with increased risk of both gastric NETs and adenocarcinomas [21, 22]. These factors together with the fact that a proportion of gastric carcinomas display markers of NE and ECL cell differentiation, suggestive of ECL cell origin, result in gastrin becoming of interest in gastric carcinogenesis. We wished to explore this further by examining the expression of CCKBR in gastric cancer, including neuroendocrine neoplasms (NENs) and gastric carcinomas of the diffuse and intestinal types. Our hypothesis was that CCKBR is expressed in ECL cell hyperplasias and NENs, and we wanted to see if the same was the case for adenocarcinomas. By assessing whether the cancer cells express CCKBR or not, one can determine which patients may experience benefits from treatment with a CCKBR antagonist.

## Materials and Methods

### Patients

Thirty-seven tumors from 37 different patients who were either treated with a total or subtotal gastrectomy or who had their tumor removed via endoscopy at St. Olavs Hospital—Trondheim University Hospital between 1995 and 2016 were included in the study. The patients were identified from previous studies [7] and by going through our records at the pathology and gastroenterology departments. All the patients were either diagnosed with adenocarcinoma or NEN. Archival formalin-fixed, paraffin-embedded (FFPE) tissue was re-examined and further subdivided into adenocarcinoma of intestinal or diffuse type according to Laurén [2], or neuroendocrine tumor (NET) grade 1 or 2, or neuroendocrine carcinoma (NEC) according to the WHO classification from 2010 [23]. American Joint Committee on Cancer (AJCC), Cancer Staging Manual, eight edition was used in order to stage the tumor [24]. Tumor size was when available noted and was defined as the area with greatest diameter recorded in the pathology specimen.

Immunohistochemical (IHC) stainings with antibodies against CCKBR, chromogranin A (CgA) and synaptophysin were performed, and in situ hybridization (ISH) with probes against CCKBR, histidine decarboxylase (HDC) and CgA was also carried out. By conducting these studies, we wished to find out whether all carcinomas of the human stomach express CCKBR and also whether all cells expressing CCKBR are positive for one or more NE markers.

### Histopathology and Immunohistochemistry

IHC was performed as described previously with a few minor modifications [25]. For this study, the following antibodies were used: chromogranin A (M0869, Dako, Glostrup, Denmark, 1:200), Synaptophysin (M7315, Dako, 1:200)*,* CCKBR (PA5-33389, Thermo Fischer Scientific, Rockford, USA, 1:250), Pepsinogen II (Ab9013-1; Abcam, Cambridge, UK, 1:1000), and HKATPase (MA3-923; Affinity Bioreagents, Golden, CO, USA, 1:1500). Mouse Link (K8021, Dako) was used with the monoclonal mouse antibodies (CgA and synaptophysin) to further amplify the signal. CgA, synaptophysin, CCKBR, and HKATPase were visualized by using an EnVision-HRP kit with DAB+ (K5007, Dako). The sections were incubated with EnVision for 30 min. Pepsinogen II was visualized using a biotinylated secondary antibody for 30 min (Biotinylated anti-sheep IgG (H + L), Vector Laboratories, Burlingame, USA, 1:150), followed by application of “Vectastain ABC kit” (Vector Laboratories) for 30 min. All sections were developed using DAB+, before counterstaining with Mayer’s haematoxylin (Sigma Life Science, Saint Louis, USA) for 10 s. Mouse IgG2b (X0944, Dako*)* was used as a negative isotype control for CgA, mouse IgG1 (X0931, Dako) was used for synaptophysin, and diluent was used as a negative control for CCKBR. Tris/EDTA (pH 9) was used as epitope retrieval solution for CCKBR, CgA and synaptophysin, and citrate buffer (pH 6) for Pepsinogen II and HKATPase. Between all steps after incubation with primary antibody a wash buffer solution was used to wash the sections. A TNT wash buffer (based on 0.1 M Trizma hydrochloride, 0.15 M NaCl and 0.05% Tween 20 (VWR, Briare, France), pH 7.5) was used on sections incubated with CCKBR, CgA and synaptophysin, and PBS-TrX wash buffer solution (consisting of phosphate-buffered saline and 0.25% Triton-X-100 (VWR, Briare, France)) was used for sections incubated with pepsinogen II and HKATPase. A known NET of the stomach was used as positive control tissue for CgA, synaptophysin and CCKBR. The surrounding connective tissue and smooth muscle tissue were used as a negative control.

### In Situ Hybridization

In situ hybridization (ISH) was done on all the tissue examined in this study by using either a single ISH assay or duplex ISH assay. In the single assay, a probe against CCKBR was used, and in the duplex assay probes against CCKBR, CgA and HDC were used. ISH with duplex ISH assay using probes against CCKBR and CgA was performed on all 37 tumors, whereas probes against CCKBR and HDC were used on only 23 tumors, due to lack of HDC probe for all 37 sections. A single ISH assay using a probe against CCKBR was used in order to ensure that the positive results found when using the ISH duplex assay were true, and not due to interference from the second probe.

For ISH RNAscope 2.5 HD Assay-Brown and RNAscope 2.5 HD duplex assay were used. The method was executed according to the protocols provided by Advanced Cells Diagnostics (ACD) with a few modifications. All steps till incubation of the probes were similar in the single and duplex assays. For both assays, 4-μm thick sections were cut from tissue blocks of paraffin and transferred to SuperFrost Plus slides. After drying the slides at room temperature overnight, they were the following day placed in a heat cabinet at 60 °C for 60 min, before being deparaffinized in NeoClear for 2 × 10 min followed by dehydration in 100% EtOH (absolute alcohol) for 2 × 2 min. The sections were then dried for 5 min before being incubated with hydrogen peroxide for 10 min at room temperature. The sections were thereafter immersed in boiling Target Retrieval (for RNA retrieval) for 15 min, before being cooled in distilled water (30 s), followed by immersion of slides in fresh 100% EtOH. After air-drying the sections for 5 min, an Immedge ™ hydrophobic barrier pen was used to draw a barrier around the tissue, followed by incubation with Protease Plus (for protein digestion) for 15 min at 40 °C. After this step, the sections were rinsed in distilled water before being incubated with the various probes for 2 h at 40 °C. For the RNAscope 2.5 HD Assay-Brown single assay target probe against human CCKBR (Probe-Hs-CCKBR, catalog number: 31101, ACD, USA), positive control probe (positive control probe–Hs-PPIB, catalog number: 313901, ACD, USA) and negative control probe (negative control probe-DapB, catalog number: 310043, ACD, USA) were used. For RNAscope 2.5 HD duplex assay target probes against human CCKBR (Probe-Hs-CCKBR-C2, catalog number: 31101-C2, ACD), CgA (Probe-Hs-CHGA, catalog number: 31111, ACD) and HDC (Probe-Hs-HDC, catalog number: 311441, ACD) were used. Positive control probe (Duplex positive control probe-Hs-PPIB-C1/POLR2A-C2, catalog number: 321641, ACD, USA) and negative control probes (2-plex negative control probe, catalog number: 320751, ACD, USA) for the duplex kit were also used. The sections going through RNAscope 2.5 HD single Assay-Brown were incubated with only a probe against CCKBR for 2 h, whereas the sections being processed with RNAscope 2.5 HD duplex assay, were incubated with a solution consisting of one part CCKBR probe in the C2 channel to 50 parts of either CgA or HDC in the C1 channel, also for 2 h. After incubation, the sections were rinsed in a wash buffer solution following the kit, and signal amplification was done according to recommendations from ACD.

The tissue sections incubated with only one probe (CCKBR) were then incubated with Amp 1 (preamplifier) for 30 min at 40 °C, Amp 2 (background reducer) for 15 min at 40 °C, Amp 3 (amplifier) at 40 °C, Amp 4 (label probe) for 15 min at 40 °C, Amp 5 for 30 min at room temperature, and finally, Amp 6 for 15 min at room temperature. After each of the steps, the sections were rinsed in wash buffer. For signal detection a DAB mixture (following the kit) was used, and counterstaining was done by staining the sections with hematoxylin and mounted using glycerel (glycergel mounting medium, DAKO).

Tissue sections incubated with two different probes (either CCKBR and CgA, or CCKBR and HDC), were subsequently incubated with Amp 1 for 30 min at 40 °C, Amp 2 for 15 min at 40 °C, Amp 3 for 30 min at 40 °C, Amp 8 for 15 min at 40 °C, Amp 5 for 30 min at room temperature, Amp 6 for 15 min at room temperature, followed by signal detection giving a red signal (following the kit). The probes in the C1 channel (CgA or HDC) stained red. After signal detection, the sections were further incubated with Amp 7 for 15 min at 40 °C, Amp 4 for 30 min at 40 °C, Amp 9 for 30 min at room temperature, and finally Amp 10 for 15 min at room temperature. For signal detection, a mixture following the kit was used, giving the probe in the C2 channel (CCKBR) a green/blue signal. The sections were counterstained by using 50% Mayer’s hematoxylin, and mounted using VectaMount (catalog number: H-5000, Vector Laboratories).

RNA quality and background signals were evaluated by using positive and negative control probes, and positive staining was defined by the presence of dot like cytoplasmatic and/or nuclear staining that was stronger than the negative control (dapB). A known NET of the stomach was used as positive control. NE cells were used as positive internal controls for all the probes, and the surrounding connective and smooth muscle tissue was used as a negative internal control. In addition, a small biopsy from normal colonic mucosa was used as a negative control.

### Scoring and Reporting

All sections were assessed by using a bright field microscope (Olympus CX41). IHC and ISH markers were assessed by one researcher, and when there were uncertainties with regard to results, a second pathologist/researcher was consulted. The quality of the staining was also accessed by a second researcher. As recommended by ACD, a semi-quantitative scoring approach to evaluate staining results was applied for both IHC and ISH.

### Classification of Tumors

The tumors were reclassified according to the WHO classification from 2010 [23] and Laurén [2]. According to Laurén, the intestinal type is characterized by the presence of gland like structures, the diffuse type by poorly cohesive cells with no or minor gland formation, the mixed type by almost equal amounts of intestinal and diffuse types, and finally indeterminate type by undifferentiated tumor. In the present study, mixed and indeterminate types are described together. The NENs are subdivided into NETs grade 1 or 2, or neuroendocrine carcinoma (grade 3) depending on the proliferative index of these tumors.

### Classification of Markers

Reporting recommendations for tumor marker prognostic studies (REMARK) with a few modifications were followed [26]. The staining was classified as either positive or negative, and if appropriate the staining intensity (weak, moderate or strong) and number of tumor cells staining positive was also noted. Less than 2% positive cells was considered a negative or very low expression, 2–10% positive cells was low, 10–40% positive cells was medium, 40–70% was high, and more than 70% positive cells was considered a very high expression. The staining pattern for the immunohistochemical markers CCKBR and CgA was further validated by using in situ hybridization technique.

### Statistical Analysis

For calculation of median and mean values as well as range for the different parameters, IBM SPSS statistics version 22 (Chicago, IL, USA) was used. The descriptive data are presented as median or mean as appropriate. In order to look for association between the various variables, Spearman’s non-parametric test was used.

### Ethical Approval

The Regional Committee for Medical and Health Sciences Research Ethics (reference number in REK: 2016/1608) approved the study. For this study, no formal consent was required.

## Results

### Patient Characteristics

Of the 37 patients included in the study, 19 (51%) were male and 18 (49%) female. Their median age at time of diagnosis was 67 (range 32–79) years. At follow-up, 30 patients had died from their stomach cancer, and seven patients were still being followed-up according to current guidelines. Of the patients still alive five were diagnosed with NETs, the remaining two with adenocarcinoma of diffuse and intestinal type.

### Tumor Characteristics

Fifteen (41%) tumors were adenocarcinoma of diffuse type, ten (27%) of intestinal type, four (11%) were a mixture of the above (or indeterminate type), and eight (22%) were NENs. Of the NENs, four were NET grade 1, two were NET grade 2, and two were NECs (grade 3). Twelve tumors (32%) were located in the cardia/fundus area, 18 (49%) in the corpus, and seven (19%) in the antrum. Of the adenocarcinomas located to oxyntic mucosa (fundus and corpus) seven of 22 (32%) were intestinal type, 11 (50%) diffuse type, and four (18%) mixed/indeterminate type. Of the antral adenocarcinomas, three of seven (43%) were of the intestinal type and four (57%) of the diffuse type. Tumor size was defined as the greatest diameter recorded in the pathology specimen, and the mean tumor size among the 37 tumors was 57 (range 2–240) mm with a SD of 48 mm. When subdivided into the two subgroups adenocarcinoma and NEN, mean tumor size was as follows: adenocarcinoma of diffuse, intestinal type or mixed/indeterminate type: 67 (range 11–240) mm with a SD of 49 mm, and for NENs: 21 (range 2–75) mm, with a SD of 24 mm. In accordance with American Joint Committee on Cancer (AJCC), Cancer Staging Manual, eight edition one (3%) out of 29 adenocarcinomas was in the Tis category, one (3%) was T1, one (3%) was T2, 22 (76%) were T3 and four (14%) were T4. Of the NENs, three of eight (38%) tumors were in the T1 category, three (38%) T2 and two (25%) T4. As an incidental finding, a gastrointestinal stromal tumor (GIST) was found in the stomach wall of case 26.

### Immunohistochemistry

All 37 tumors were examined with antibodies against CgA, synaptophysin, and CCKBR. The staining of CgA was cytoplasmatic, and the staining intensity was either strong or non-existent. Synaptophysin was expressed in the cytoplasm of the positive cells, and the staining intensity of synaptophysin was strong in the NETs, and weaker in the NECs and adenocarcinomas. The staining of CCKBR was mainly cytoplasmatic, and the staining intensity mainly moderate for NENs, and weak to moderate in the adenocarcinomas. The largest number of CCKBR expressing tumor cells was seen among the NENs. In addition to staining tumor cells and scattered NE cells, weak and possible non-specific binding was seen in luminal epithelial cells, fibroblast-like stromal cells, and endothelial cells.

### Neuroendocrine Neoplasms

As NENs are known to express NE markers and CCKBR, these are considered a gold standard and described separately from the adenocarcinomas. All eight (100%) NENs expressed CgA and synaptophysin, and seven (88%) expressed CCKBR. The one case negative for CCKBR was NEC. The staining intensity was strong for the NE markers and moderate for CCKBR.

### Adenocarcinomas (Intestinal, Diffuse, and Mixed/Indeterminate Types)

Eight of 29 (28%) adenocarcinomas expressed CgA. This marker was observed in one of ten (10%) intestinal type adenocarcinoma, five of 15 (33%) diffuse type adenocarcinoma, and two of four (50%) mixed/indeterminate type. Expression of synaptophysin was observed in nine of 29 (31%) adenocarcinomas, and of these the marker was expressed in three of ten (30%) intestinal type adenocarcinomas, four of 15 (27%) diffuse type adenocarcinomas, and two of four (50%) mixed/indeterminate type adenocarcinomas. CCKBR was expressed in 26 of 29 (90%) adenocarcinomas, where positive cases were observed in nine of ten (90%) intestinal type adenocarcinomas, 14 of 15 (93%) diffuse type adenocarcinoma, and three of four (75%) mixed/indeterminate type adenocarcinomas. Table 1 gives an overview of staining results including approximately how many cells are positive for the various antibodies in each case.

**Table 1:** Staining results from 37 cases of stomach cancer. The amount of positive cells is given values ranging from 0 to 4, where 0 indicates < 2% positive cells, 1 is 2–10% positive cells, 2 is 10–40% positive cells, 3 is 40–70% positive cells, and 4 is > 70% positive cells

Case	Age	Gender	Tumor type	Synaptophysin	Chromogranin A		HDC	CCKBR	
				*IHC*	*IHC*	*ISH*	*ISH*	*IHC*	*ISH*
1	69	Female	NET	4	2	4		2	4
2	66	Male	NET	4	4	4	2	4	4
3	52	Female	NET	4	4	4	4	4	4
4	49	Female	NET	4	4	4	4	4	4
5	32	Male	NET	4	4	4		1	4
6	72	Male	NET	4	4	4		4	4
7	77	Female	NEC	3	2	1	1	3	2
8	76	Female	NEC	4	3	4	2	0	4
9	65	Male	Intestinal	0	0	1	0	2	1
10	70	Male	Intestinal	2	0	1	1	2	1
11	72	Male	Intestinal	0	0	1	0	2	1
12	74	Male	Intestinal	0	0	1	0	3	3
13	59	Male	Intestinal	0	0	0	1	2	0
14	57	Male	Intestinal	0	0	1	0	0	1
15	58	Male	Intestinal	2	0	0	0	2	0
16	50	Male	Intestinal	0	0	0	0	1	0
17	59	Male	Intestinal	0	0	0	0	2	0
18	74	Female	Intestinal	1	1	1		1	0
19	67	Female	Diffuse	0	0	0	0	1	0
20	69	Female	Diffuse	0	0	1	1	2	0
21	77	Male	Diffuse	1	2	2		3	2
22	73	Female	Diffuse	2	2	2	1	2	1
23	76	Female	Diffuse	0	0	1		1	0
24	79	Male	Diffuse	0	0	0		1	0
25	56	Female	Diffuse	0	0	1		0	0
26	72	Female	Diffuse	1	1	1		1	3
27	44	Female	Diffuse	1	1	1		3	0
28	67	Male	Diffuse	0	0	0	0	1	1
29	56	Male	Diffuse	0	1	1		1	0
30	74	Male	Diffuse	0	0	0	0	2	0
31	48	Female	Diffuse	0	0	1	0	1	0
32	71	Female	Diffuse	0	0	1		1	0
33	49	Female	Diffuse	0	0	1		2	1
34	56	Male	Mixed	0	0	2	1	0	0
35	52	Male	Mixed	0	0	0	1	1	0
36	70	Female	Mixed	2	2	2		1	1
37	78	Female	Mixed	2	2	2	0	2	3

*NET* neuroendocrine tumor, *NEC* neuroendocrine carcinoma, *Intestinal* adenocarcinoma of intestinal type according to Laurén, *Diffuse* adenocarcinoma of diffuse type according to Laurén, *Mixed* adenocarcinoma of mixed or indeterminate type, according to Laurén

### Gastric Mucosa

In some of the tumor-containing sections, gastric mucosa was also visualized. The morphology ranged from normal gastric mucosa to CAG with varying degrees of ECL cell hyperplasia. In the mucosa with changes consistent with CAG, CCKBR expression was only observed in ECL cells. In two separate biopsies, one from a patient with normal gastric mucosa and one from a patient with Zollinger-Ellison syndrome, CCKBR were observed in most cells below the isthmus area of the glands. The expression of CCKBR was strongest in the NE cells. HE stained sections and further examination with HKATPase and pepsinogen II demonstrated parietal cells and chief cells to be among the cells found in the same location.

### In Situ Hybridization

ISH with a duplex ISH assay using a probe against CCKBR and CgA was performed on all 37 tumors, whereas a probe against CCKBR and HDC was used on only 23 tumors. CCKBR mRNA stained green/blue and CgA mRNA and HDC mRNA stained red. The sections were viewed in a bright field microscope, and proportion of cells expressing green/blue and red signals was noted. For all probes used, the staining intensity/number of dot-like structures seen in each cell was higher in NENs compared with carcinomas. HDC was in addition to staining tumor cells positive in some stromal cells, which were thought to represent mast cells.

Interestingly, in ECL cell hyperplasia and NETs there was co-expression of CCKBR and CgA or HDC in almost all normal ECL- cells and ECL-derived tumor cells. There was also some degree of co-expression in adenocarcinoma of the diffuse and intestinal types, but to a much lesser extent than the NETs.

### Neuroendocrine Neoplasms

**Fig. 1:**
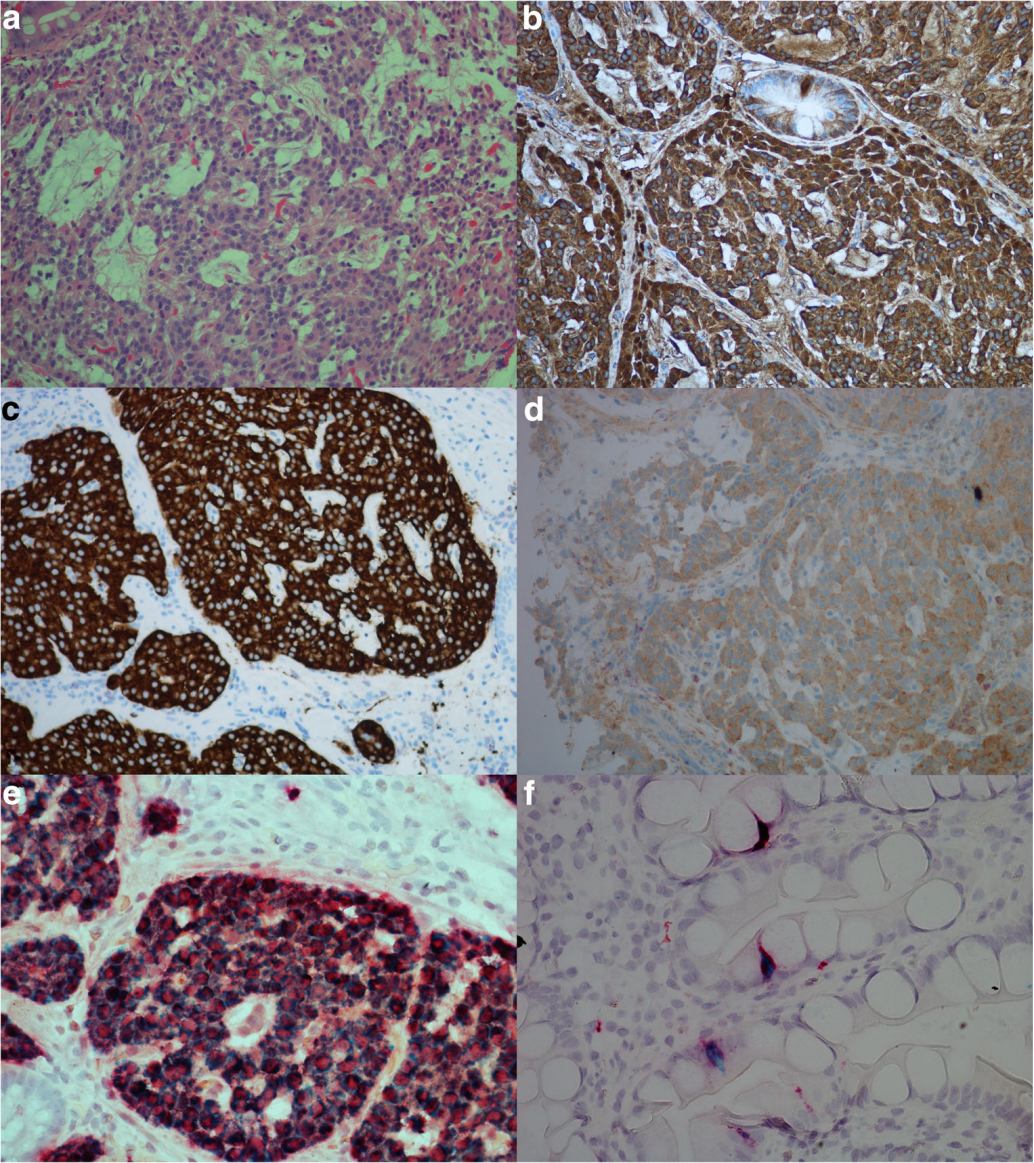
Case 6, neuroendocrine tumor grade 1 in a patient with chronic atrophic gastritis illustrated by **a** Hematoxylin and eosin, × 20, and by positive expression for **b** chromogranin A, × 20, **c** synaptophysin, × 20, **d** cholecystokinin B receptor, × 20, **e** in situ hybridization with two probes, illustrating co-expression of cholecystokinin B receptor (green/blue) and chromogranin A (red), × 40, **f** in situ hybridization with dual expression of cholecystokinin B receptor and chromogranin A in neuroendocrine cells (blue/green = cholecystokinin B receptor, red = chromogranin A, black = dual expression), × 40

**Fig. 2:**
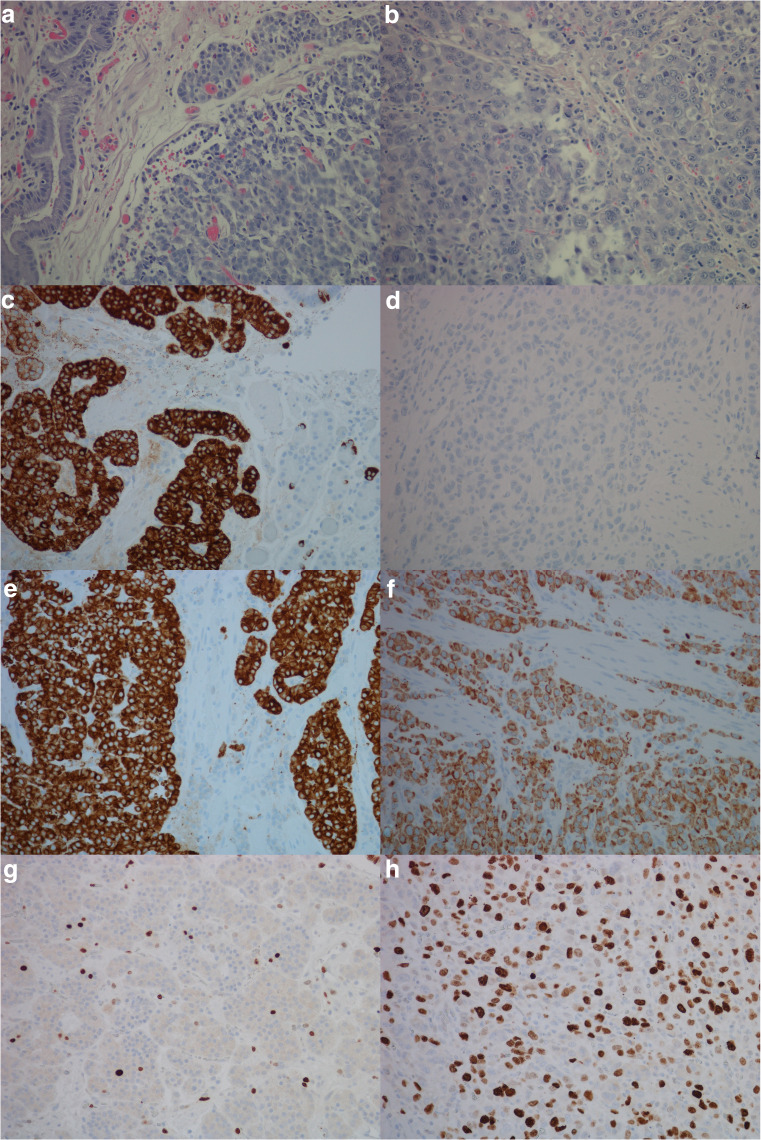

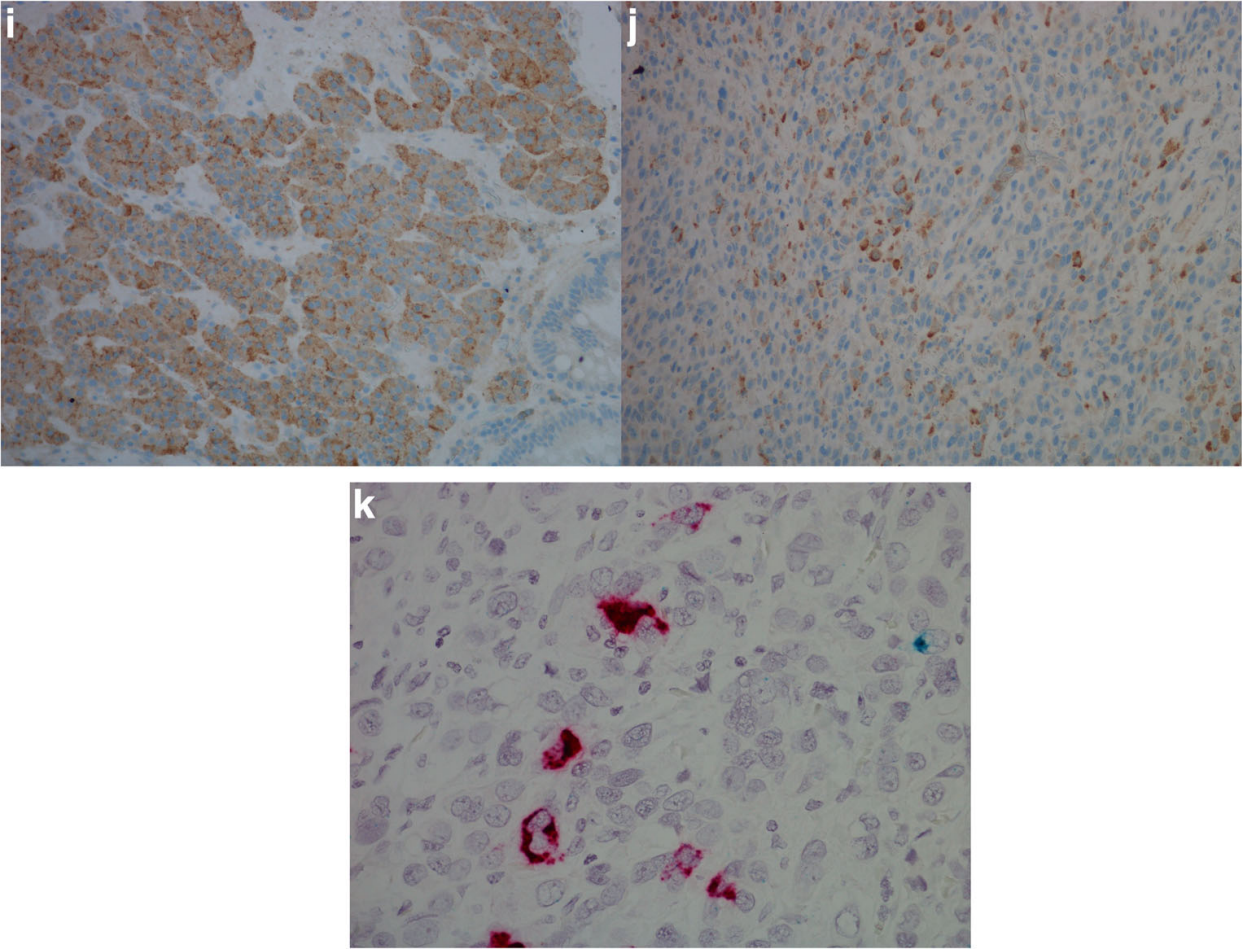
Case 7, patient with enterochromaffin-like cell hyperplasia, neuroendocrine tumor and neuroendocrine carcinoma illustrated by, **a** hematoxylin and eosin, neuroendocrine tumor, × 20, **b** hematoxylin and eosin, neuroendocrine carcinoma, × 20, **c** chromogranin A positive neuroendocrine tumor, × 20, **d** chromogranin A negative neuroendocrine carcinoma, × 20, **e** synaptophysin positive neuroendocrine tumor, × 20, **f** synaptophysin positive neuroendocrine carcinoma, × 20, **g** Ki67 expression in 2–5% of tumor cells, neuroendocrine tumor, × 20, **h** Ki67 expression in > 20% of tumor cells, neuroendocrine carcinoma, × 20, **i**) cholecystokinin-B receptor protein expression, neuroendocrine tumor, × 20, **j**) cholecystokinin-B receptor protein expression, neuroendocrine carcinoma × 20, **k**) in situ hybridization, cells with dual expression of cholecystokinin-B receptor and chromogranin A in neuroendocrine carcinoma (red/black/green), × 40. Areas of co-localization appear black

Expression of CgA mRNA and CCKBR mRNA was noted in all eight (100%) NENs examined. Only five cases were investigated with probe against HDC, all of these cases positive. For all probes, the highest number of dot-like structures was observed in NETs and the lowest number in NECs. The results of IHC and ISH are illustrated in Fig. 1. Interestingly, in case 7 (Fig. 2), all the stages from ECL cell hyperplasia, NET and NEC were observed, and further examination with IHC and ISH clearly demonstrated positivity for both CgA and CCKBR in all the various components of the tumor. The number of positive tumor cells in the less differentiated areas of the tumor was, however, low indicating dedifferentiation of the tumor tissue. Synaptophysin, on the other hand, was positive in most of the tumor cells. This tumor was originally diagnosed as indeterminate or diffuse type adenocarcinoma, partly due to IHC not being performed.

### Adenocarcinomas (Intestinal, Diffuse, and Mixed/Indeterminate Types)

**Fig. 3:**
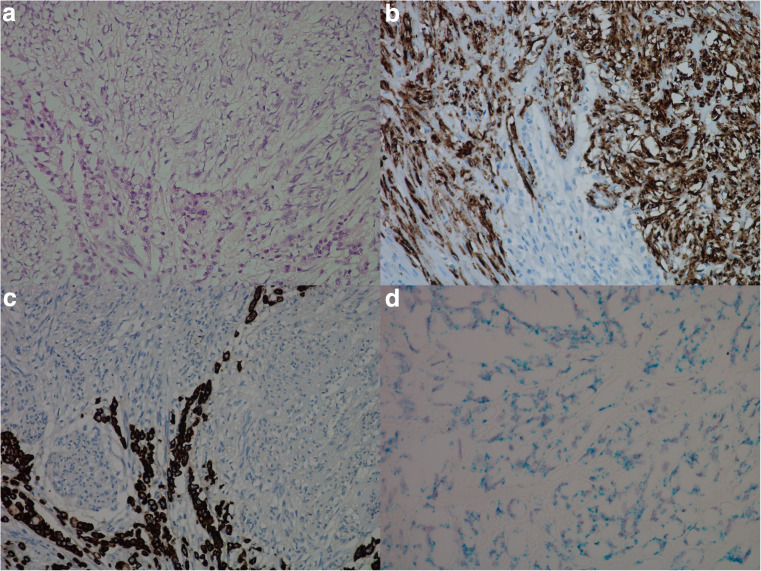
Case 26. Gastrointestinal stromal tumor (GIST) with carcinoma cells of diffuse type in the periphery illustrated by **a** hematoxylin and eosin, × 20, **b** positive expression for CD117 protein, × 20, **c** pancytokeratin expression in carcinoma cells just next to GIST, × 20, **d** in situ hybridization, expression of CCKBR mRNA (green/blue) in GIST, × 40

**Fig. 4:**
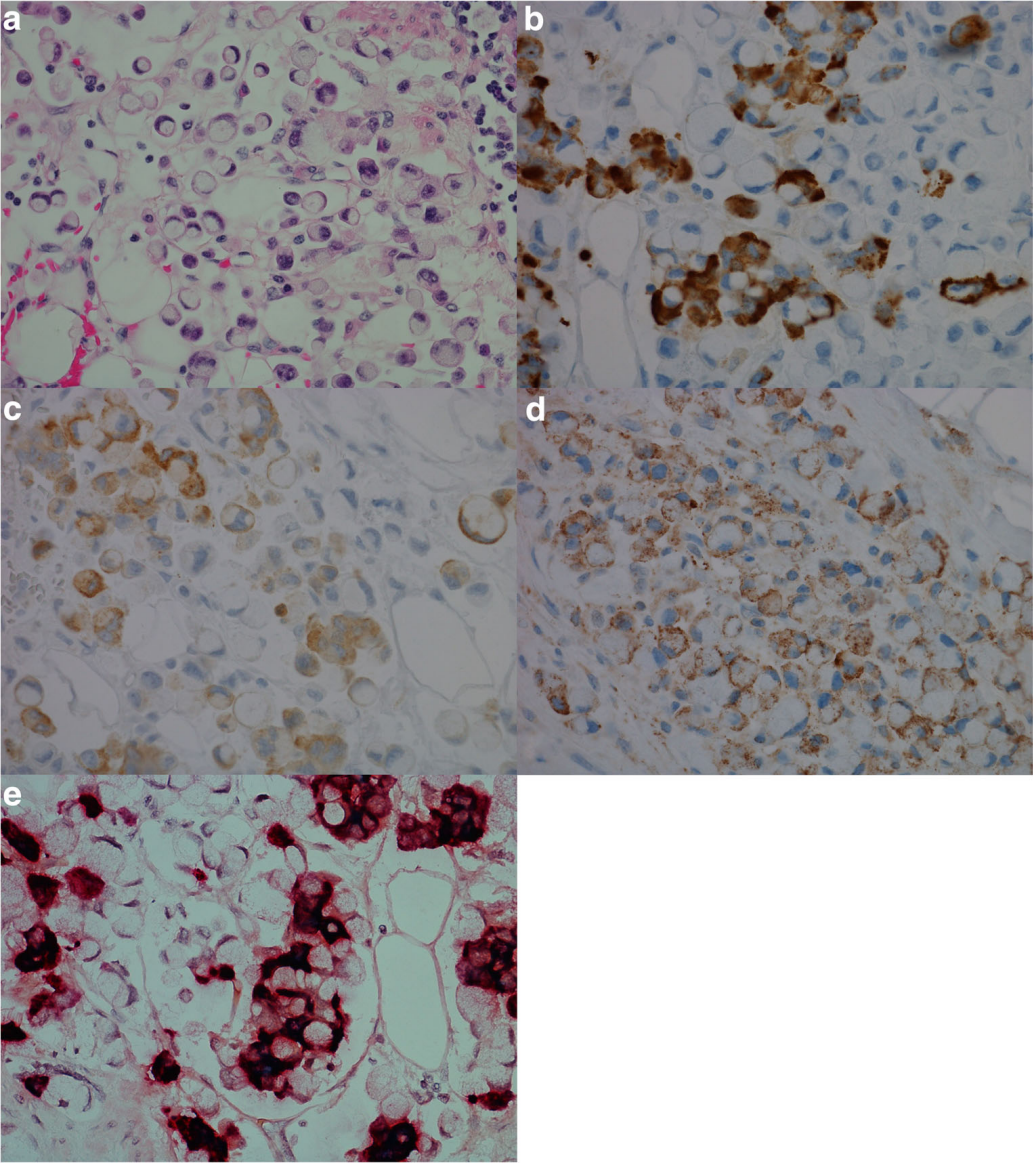
Case 21. Adenocarcinoma of diffuse type illustrated by **a** hematoxylin and eosin, × 40, **b** with chromogranin A (protein) positive cells, × 40, **c** synaptophysin (protein) positive cells, × 40, **d** cholecystokinin-B receptor (protein) positive cells, × 40, and **e** in situ hybridization with dual expression of chromogranin A mRNA (red) and CCKBR mRNA (green/blue), × 40 (areas of co-localization appear black)

CgA mRNA was observed in 20 of 29 (69%) adenocarcinomas, where six of ten (60%) intestinal type, 11 of 15 (73%) diffuse type, and three of four (75%) mixed/indeterminate type were positive. Of the 18 adenocarcinomas examined for HDC mRNA, two of nine (22%) intestinal type, two of six (33%) diffuse type, and two of three (67%) mixed/indeterminate type were positive. When examined for CCKBR mRNA 12 of 29 (41%) adenocarcinomas expressed this marker, where five of ten (50%) intestinal types, five of 15 (33%) diffuse types and two of four (50%) mixed/indeterminate types expressed this marker. As an incidental finding, a GIST was observed in the stomach wall of case 26 (Fig. 3). This tumor was surrounded by mostly CCKBR negative carcinoma cells, but the GIST itself expressed copious amounts of CCKBR mRNA. A summary of staining results is found in Table 1, and illustration of results is found in Figs. 3 and 4.

### Normal Gastric Mucosa

**Fig. 5:**
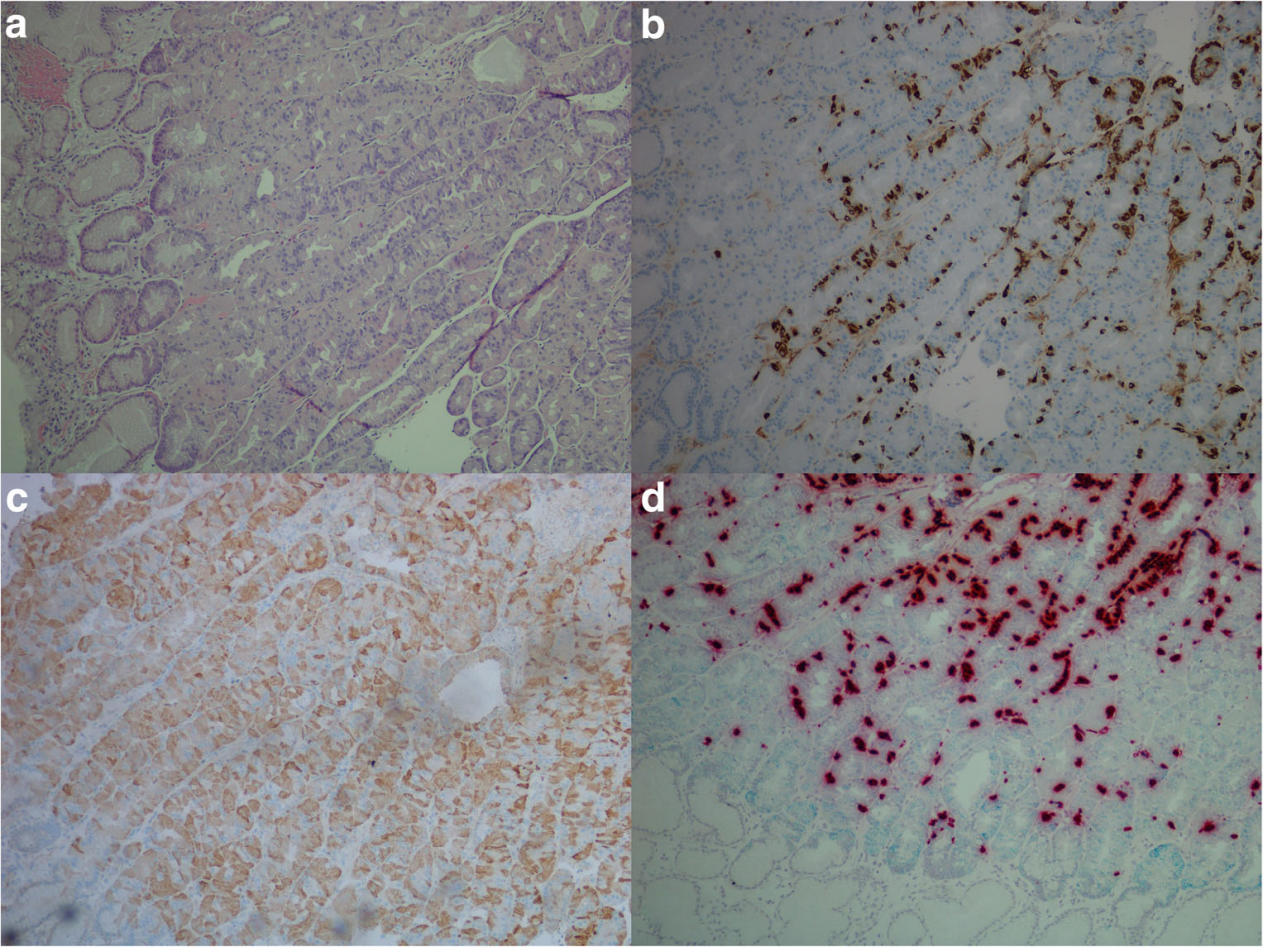
Normal gastric mucosa from a patient with Zollinger-Ellison syndrome illustrated by **a** hematoxylin and eosin, × 10, **b** chromogranin A (protein) positive neuroendocrine cells, × 10, **c** cholecystokinin-B receptor (protein) expression in normal gastric mucosa, × 10, and **d** in situ hybridization (duplex) illustrating dual expression of cholecystokinin-B receptor mRNA (green/blue) and chromogranin A mRNA (red) in neuroendocrine cell (dual expression appearing black), and cholecystokinin-B receptor positive cells in other epithelial cells below the isthmus of the gastric gland

When possible, gastric mucosa in close proximity to tumor was examined. In addition, separate biopsies from two patients, where one was from a patient with normal gastric mucosa and the other from a patient with Zollinger-Ellison syndrome, were also investigated. In the mucosa from the patient with Zollinger-Ellison, hyperplasia of ECL cells was observed. CCKBR mRNA was expressed in ECL cells and faintly in glandular cells below the isthmus area. The number of dot-like structures was higher in the ECL cell when compared to the other cell types. Further IHC investigations on normal gastric mucosa with antibodies against HKATPase and pepsinogen II showed parietal and chief cells to be among the weak CCKBR mRNA expressing cells (Fig. 5). This was not seen to the same extent in larger sections or in sections where the normal epithelium was replaced by intestinal metaplasia.

### Characteristics of Patients Expressing CCKBR mRNA

Of the 29 adenocarcinomas examined, 12 tumors expressed CCKBR mRNA, and 11 of these tumors co-expressed CgA mRNA. Of the 12 CCKBR mRNA expressing tumors, five (42%) were from women and seven (58%) from men. Five (42%) were adenocarcinoma of intestinal type, five (42%) of diffuse type and two (17%) of mixed/indeterminate type. Four tumors were located to the cardia/fundus region, five (42%) to the corpus and three (25%) to the antrum.

### Correlations

When considering all 29 adenocarcinomas, there was a strong association between CgA protein and synaptophysin protein (*r* = 0.7, *p* < 0.01), and CgA protein and CgA mRNA (*r* = 0.6, p < 0.01)). There was a moderate, but significant association between CCKBR mRNA and CgA mRNA (*r* = 0.5, *p* < 0.01). When considering the group consisting of CCKBR mRNA expressing tumors, a positive association between the expression of CgA protein and CgA mRNA was observed (*r* = 0.9, *p* < 0.01). The association between CCKBR protein and CCKBR mRNA was weak and not statistically significant (*r* = 0.3, *p* = 0.08).

## Discussion

Our study, which we consider a qualitative study, demonstrates that CCKBR mRNA and CCKBR protein are expressed in normal ECL cells, ECL cell hyperplasia and NENs. Among the NENs, the expression of CCKBR was high in the NETs, and low in NECs. This can be explained by dedifferentiation of the tumor cells. Expression of CCKBR was also observed in a number of cases with adenocarcinoma of the diffuse and intestinal types, but in a lower number of tumor cells when compared with NENs. Co-expression of CCKBR mRNA and CgA mRNA and CCKBR mRNA and HDC mRNA was frequently observed, which makes sense as the NENs and even some cases of adenocarcinomas of the diffuse type are thought to originate from ECL cells [7]. There was no significant association between the expression of CCKBR protein and CCKBR mRNA, and quite a number of cases expressing CCKBR protein did not express CCKBR mRNA, which partially could be due to poor mRNA quality of the larger sections, variable fixation time and variable age and condition of the tissue blocks. The choice of antibody may be of relevance, as the antibody used in this study was a polyclonal rabbit antibody. Polyclonal antibodies are known to give more background staining compared to monoclonal antibodies, which are considered more specific. Moreover, polyclonal antibodies are not specific enough to ensure that the antibody binds exclusively to the CCKBR. Even a sequence in a monoclonal antibody directed towards the CCKBR may not be specific enough to guarantee this, as the specific sequence in question may react with other receptors with the very same epitope. The strongest CCKBR protein expression was observed in the NENs. Surprisingly though, one of the NECs (case 8) with high CCKBR mRNA expression did not express any CCKBR protein in the tumor. A GIST, which was incidentally found in the stomach wall of case 26, expressed ample amounts of CCKBR mRNA. This was not seen to the same degree in the surrounding carcinoma cells. Finding of CCKBR in GISTs, which are thought to originate from interstitial cells of Cajal, has also been described in previous study [27].

Of the adenocarcinomas located to the antrum, three were intestinal type and four of diffuse type. Further investigations revealed that six of them expressed CgA mRNA, three (two intestinal and one diffuse) expressed CCKBR mRNA, whereas none expressed HDC mRNA. A study by Choi et al. demonstrated that the antral mucosa contains a mixture of oxyntic glands and antral glands containing gastrin-producing cells [28]; thus, this may partly explain the presence of CCKBR mRNA expression in tumors originating from the antrum.

Gastrin exerts its effects by binding to CCKBR in the stomach and is involved in secretion of acid and cellular proliferation. Whether gastrin has a dominating role in the development of gastric carcinoma has been under debate. Studies on rats have revealed that hypergastrinemia results in the development of ECL-derived NETs [29, 30]. On the other hand, hypergastrinemia in insulin-gastrin (InsGas) transgenic mice causes gastric adenocarcinoma which is not accompanied by hyperplasia of ECL cells [31]. Gastrin stimulates function (histamine release) and ECL cell proliferation in a parallel way. The intracellular down-stream mechanisms for the interaction of gastrin with its receptor have been examined mainly around year 2000 by transfection of the receptor to cell lines [32, 33] and in the cell with CCKBR, the ECL cell [34]. These studies showed that gastrin to CCKBR interaction results in phosphoinositol breakdown leading to a rapid Ca^2+^ release activating protein kinase D and MEK-dependent ERK activation as well as increased cyclins: all involved in the regulation of proliferation. In order to further explore the effects of gastrin on gastric cancer cells, further studies are needed. Detection of intracellular signal substances will not necessarily prove that gastrin has a positive trophic effect on gastric cancer cells; thus, biological effects of gastrin are better demonstrated by confirming an inhibitory effect of a CCKBR antagonist on such tumors in vivo. As a hormone has direct effects only on cells having the receptor, patients selected for such a trial would need to express CCKBR on their tumor cells.

A close relationship between gastric NENs and adenocarcinomas of the stomach has been described [35], and the preservation of the CCKBR on gastric cancer cells may similarly to gastric NETs make them susceptible for treatment with a CCKBR antagonist (netazepide). Hypergastrinemia is a known cause of NENs in humans. Various studies done on mastomys [36] and other rodents [29, 37] discovered the development of adenocarcinomas in the stomach after prolonged acid suppression. Mastomys are genetically predisposed to developing NETs in the stomach, which is thought to be due to an abnormality in the CCKBR itself [38, 39]. Acid suppression in this rodent further amplifies the development of NETs. The study by Soga et al. led to a reclassification of adenocarcinomas to neuroendocrine carcinomas in the mastomys. A few studies in man led to similar findings [40]. Studies done by our group have demonstrated that a proportion of human gastric adenocarcinomas express NE markers, suggesting that some of these tumors may originate from enterochromaffin like (ECL) cells [4]. By using more sensitive methods, we were able to demonstrate that a substantial number of adenocarcinomas of the diffuse type express NE markers, supporting this view [5, 41]. Interestingly, most tumors removed from patients with hypergastrinemia due to pernicious anemia expressed NE markers suggestive of ECL cell origin [42]. This finding is especially convincing among adenocarcinomas of the diffuse type [7]. The ECL cell may develop into malignant tumors by steps of ECL cell hyperplasia, neuroendocrine tumor (NET), and, finally, carcinoma [6]. Case 7 (Fig. 2) demonstrates this. From this, it is quite clear that the location of CCKBR is important for the carcinogenic process in hypergastrinemia.

The finding of NE markers in NETs and NECs is not surprising as the classification of these tumors is based on a specific morphology and/or the expression of NE markers in these tumors. As NENs in the stomach are thought to derive from ECL cells, which in turn are influenced by gastrin levels, the finding of CCKBR mRNA in these tumors is also expected. Patients with known NETs in the stomach treated with the CCKBR antagonists (netazepide) have experienced partial and even complete remission, supporting the notion that CCKBR is expressed in these tumors [14]. Furthermore, both NETs and carcinomas are associated with autoimmune gastritis and *Helicobacter pylori* (Hp) gastritis [8, 20]. Hypergastrinemia in patients with gastric carcinoma has also been reported [9, 10, 43]. Some of these tumors, especially adenocarcinomas of the diffuse type, express NE markers indicating ECL cell origin [5, 6, 42]. Therefore, the expression of CCKBR (in 90% of adenocarcinomas by IHC, and 41% by ISH) was not unexpected.

*Helicobacter pylori* infection is considered to be the main factor in gastric carcinogenesis. The role of gastrin in gastric cancer cannot, however, be overlooked as some studies have demonstrated hypergastrinemia in patients with these cancers [9, 10, 43]. Examination of CCKBR may help to tailor treatment, and if a tumor expresses CCKBR, a CCKBR antagonist may be applied. The CCKBR antagonist, netazepide, aids in preventing the formation of gastric NETs in mastomys [44]. Similar findings are observed in humans, and a reduction in both size and number of gastric NETs are observed in patients given this drug [45, 46]. The CCKBR antagonist netazepide also reduces the density of ECL cells in flat gastric mucosa in those patients with NETs due to atrophic gastritis [15].

In most of the tissue samples we examined, the normal gastric mucosa was replaced by tumor tissue or CAG. The gastric mucosa in CAG showed CCKBR mRNA in only ECL cells. In a few smaller biopsies from normal gastric mucosa and gastric mucosa from a patient with Zollinger-Ellison syndrome, weak expression of CCKBR mRNA was in addition to ECL cells noted in gastric epithelial cells below the isthmus of the glands. The amount of CCKBR mRNA was clearly higher in the ECL cells compared with other cells in the gastric mucosa. The same was observed at protein level. These findings were mainly observed in smaller and more recent biopsies, not in larger and older samples. Over-fixation and long-time storage of samples may destroy the mRNA in the tissue [47]. Various physiological studies have found the CCKBR receptor to be localized on ECL cells in the gastric corpus and fundus [48–50]. Kopin et al. were able to clone CCKBR from enriched canine parietal cells. This preparation was, however, only 95% pure still making it possible that the receptor was cloned from non-parietal cells [16]. The presence of CCKBR on the ECL cell has been undisputed since the CCKBR receptor was cloned from ECL derived NETs [51]. The cellular localization of CCKBR, on the other hand, has been disputed from the time the three major secretagogues of gastric acid secretion, acetylcholine, gastrin and histamine were discovered, and particularly the relationship between gastrin and histamine in this stimulation. Various studies established that gastrin was central in the physiological regulation of gastric acid secretion [52–56]. However, the interaction between gastrin and histamine was not settled. Black and co-workers showed that also histamine was a physiological regulator of acid secretion [53]. Berglindh and Öbrink demonstrated that a cholinergic compound as well as histamine stimulated aminopyrine uptake in glands/cells from rabbit, whereas gastrin was without any effect strongly indicating that there was no CCKBR on the parietal cell [54]. Soll, on the other hand, studied isolated canine cells from the oxyntic mucosa and reported a very faint effect by gastrin compared with a cholinergic agent and histamine [55]. Histamine is, however, much more potent in canine parietal cells compared with other species so the faint and inconsistent effect by gastrin could have been due to stimulation by histamine released from neighboring ECL cells. In fact, some years later, we found that gastrin only stimulated aminopyrine uptake in those preparations where gastrin also increased histamine concentration [56]. Shortly afterwards Prinz and co-workers described the regulation of the histamine release from isolated ECL cells [57]. The totally isolated vascularized perfused rat stomach allowed us to determine acid secretion and histamine concomitantly [48], and we could show that the histamine release was sufficient to explain the acid stimulation by gastrin [58]. Finally, Athman and co. workers studied rabbit isolated oxyntic glands using confocal microscopy to assess intracellular Ca^2+^ concentration [59]. They found that gastrin-stimulated increase in intracellular Ca^2+^ in parietal cells could be blocked by a histamine-2 blocker up to a gastrin concentration of 1000 pM, but when increasing the gastrin concentration further ten times, the histamine-2 blocker could no longer block the effect [59]. As we see it, this does not indicate a separate CCKBR on the parietal cell, but more likely interference with a structurally related receptor on the parietal cell. Rangachari and McWade studied canine isolated oxyntic mucosa in Ussing chambers and found that pentagastrin in contrast to histamine stimulated acid secretion inconsistently, and more importantly very short-lived which could be due exhaustion of stored histamine [60]. The trophic effects of gastrin are also a part of the functional effects of gastrin. Gastrin has a selective trophic effect on the ECL cell and a general effect on the other oxyntic mucosal cells [61]. The general effect can be explained by stimulation of the stem cell either by a CCKBR there or indirectly by a mediator released from the ECL cell for instance Reg protein [62]. For inducing an effect, gastrin has to bind to its receptor. Binding studies will therefore reflect possible biological effects [63]. During the last decades, there have been many attempts to localize the CCKBR by immunohistochemistry [64–66], but there have been problems due to lack of specificity of the antibodies, presumably due to binding to structurally related receptors. In the present study, we have tried to improve the specificity by doing in situ hybridization as well, but even constructed probes may not be completely specific. A Danish study assessed CCKBR mRNA by real time PCR and receptor protein by western blotting in 20 gastric adenocarcinomas and found both in all tumors [67]. To conclude the question of the localization of CCKBR in normal stomach, the results have been divergent, but we will underline that functional results probably are the most reliable ones. Therefore, the receptor is localized to the ECL cell without doubt, whereas other locations are uncertain.

## Conclusion

In summary, we found that all NENs expressed NE markers and CCKBR. The expression of these markers were, however, lower in NECs compared with the NETs probably due to dedifferentiation of the tumor. The same markers were also expressed in a proportion of adenocarcinomas of the diffuse and intestinal type, incriminating gastrin in the development of gastric cancer. Thus, targeting the CCKBR may be an option when treating carcinomas of the stomach, and perhaps this may improve the now dismal prognosis of this disease. Therefore, the determination of CCKBR (gastrin receptor) in gastric carcinoma cells will help to select patients for treatment trials with netazepide and is also important for the understanding of gastric carcinogenesis.
